# Antioxidant N-Acetylcysteine Facilitates Breast Cancer Metas-Tasis via Immunosuppressive Reprogramming of Neutrophils

**DOI:** 10.3390/ijms27010526

**Published:** 2026-01-04

**Authors:** Jiawen Zhang, Di Wang, Huige Wang, Qiuyu Wu, Menghao Liu, Qing Li, Zheng Gong

**Affiliations:** 1 School of Basic Medical Sciences, Center for Big Data and Population Health of IHM, Anhui Medical University, Hefei 230032, China; 2 Institute of Health and Medicine, Hefei Comprehensive National Science Center, Hefei 230601, China; 3 Joint Research Center for Food Derived Functional Factors and Synthetic Biology of IHM, School of Food and Biological Engineering, Hefei University of Technology, Hefei 230009, China; 4 Key Laboratory of Experimental Teratology, Ministry of Education, Institute of Molecular Medicine and Genetics, School of Basic Medical Sciences, Cheeloo College of Medicine, Shandong University, Jinan 250012, China; 5 State Key Laboratory of Immune Response and Immunotherapy, Institute of Health and Medicine, Hefei Comprehensive National Science Center, Hefei 230027, China

**Keywords:** N-acetylcysteine, lung metastasis, neutrophils, immunosuppression, breast cancer

## Abstract

N-acetylcysteine (NAC) is a widely used antioxidant. It has also attracted significant research interest with regard to its role in cancer progression, although the mechanisms involved remain controversial and poorly understood. Here, using murine models of breast cancer metastasis, we found that systemic NAC administration significantly enhanced pulmonary metastasis without altering primary tumor growth in immunocompetent mice, whereas this metastasis-promoting property of NAC was abrogated in T cell-deficient mice. This phenomenon was not due to the direct effects of NAC on T cells or tumor cells, since in vitro studies indicated that NAC exhibited no impact on the effector functions of T cells or the malignant behavior of breast cancer cells. Mechanistically, we demonstrated that NAC endows neutrophils with an immunosuppressive phenotype, which is characterized by the upregulation of immunosuppressive genes, and these NAC-educated neutrophils potently suppress the activation and effector functions of T cells. Collectively, our study reveals a previously unrecognized role played by NAC in regulating breast cancer lung metastasis by orchestrating the myeloid-dependent suppression of anti-tumor T cell immunity and suggests a need to consider immune-mediated mechanisms when evaluating the systemic impact of antioxidant agents in cancer patients.

## 1. Introduction

Antioxidants are widely present in food, dietary supplements, and pharmaceuticals [1]. Through donating electrons, antioxidants can neutralize free radicals and reactive oxygen species (ROS) to prevent cellular damage and pro-tumorigenic signaling [2,3]. Numerous studies have demonstrated the anti-tumor effects of antioxidants in both in vitro and in vivo contexts, mainly by suppressing cell growth, promoting apoptosis, and inhibiting epithelial–mesenchymal transition [4,5,6,7,8,9]. However, this prevailing perspective has been challenged in recent years. Some studies have shown that antioxidants may exert tumor-promoting effects. For instance, low concentrations of vitamin C promoted the growth of melanoma cells [10]. Vitamin E accelerated tumor progression in a murine model of colorectal cancer [11]. A human clinical study revealed that participants receiving antioxidants had a significantly increased risk of prostate cancer [12].

By providing cysteine, NAC serves as a critical precursor in the biosynthesis of glutathione (GSH), which is one of the most powerful intracellular antioxidants [13]. In addition, NAC is widely used as a potent mucolytic agent in clinical settings to reduce the viscosity and elasticity of airway mucus [14]. Previous studies have indicated a complex role played by NAC in modulating cancer progression. It has been shown that NAC accelerates the proliferation of lung tumor cells by reducing p53 activation [15]. Other research demonstrated that chronic NAC treatment induced lung adenocarcinoma in aging mice due to the inactivation of JunD [16]. Additionally, NAC promoted invasive properties of melanoma cells by increasing GSH levels and activating Ras homolog family member A (RhoA) [17]. However, studies in MYC-driven B lymphoma cells suggested that NAC induced cancer cell apoptosis and repressed tumor growth [18]. In contrast to the well-established roles of NAC in regulating the malignant behavior of cancer cells, its effects on immune cells are less well characterized.

The vast majority of cancer-related mortality is caused by the distant metastasis of tumors [19]. The lung is one of the most frequent sites of metastasis for malignancies. Limited evidence has shown that certain food additives and dietary components, including palmitic acid, vitamin E, and taurine, can potentially regulate lung metastasis [20,21,22]. In this work, we showed that the antioxidant NAC facilitates lung metastasis in mouse breast cancer models. Mechanistically, NAC did not promote the malignant behavior of in vitro cultured breast tumor cells but instead endowed neutrophils with an immunosuppressive phenotype, thereby dampening the anti-tumor functions of T cells. Our work, therefore, revealed a previously unrecognized role played by NAC in regulating breast cancer lung metastasis and identified a novel mechanism controlling this process.

## 2. Results

### 2.1. Antioxidant NAC Facilitates Lung Metastasis of Breast Cancer

**Figure 1:**
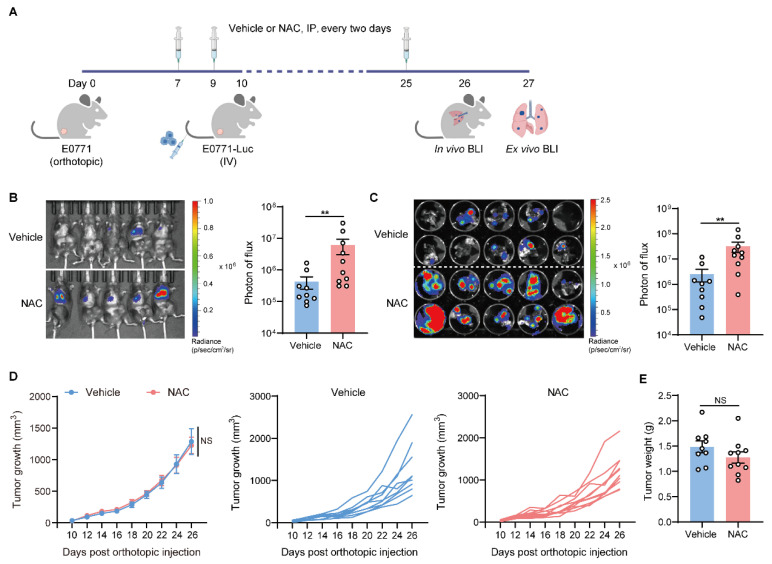
NAC facilitates lung metastasis of breast cancer. (**A**–**E**) As depicted in the schematic (**A**), female mice first received an orthotopic inoculation of 2 × 10^5^ E0771 cells. From day 7, mice were intraperitoneally (IP) injected with vehicle or NAC (150 mg/kg) every 2 days. On day 10, the luciferase-labeled E0771 cells (E0771-Luc) were intravenously (IV) injected (1 × 10^6^ cells) into the tumor-bearing mice. On day 26, lung metastasis was quantified via in vivo BLI (**B**). On day 27, lung tissues were excised for ex vivo BLI (**C**). The primary tumor volume (**D**) was measured from day 10 to day 26 post orthotopic implantation of E0771 cells, and tumor weight (**E**) was determined on day 27 (*n* = 9–10). *n* represents the number of biological replicates. Data are shown as mean ± SEM, and statistical significance was determined using the Mann–Whitney test (**B**,**C**,**E**) or two-way ANOVA (**D**). ** *p* < 0.01; NS, not significant.

To investigate the effects of NAC on lung metastasis progression, we employed a modified experimental lung metastasis model [23], in which luciferase-labeled cancer cells were intravenously (IV) injected into mice bearing orthotopic tumors at the pre-metastatic stage (Figure 1A). NAC was intraperitoneally (IP) injected into mice every two days, and metastatic colonization was quantified via bioluminescence imaging (BLI). As shown, the administration of NAC significantly promoted tumor colonization in the lung (Figure 1B,C), while the progression of primary tumors was not affected (Figure 1D,E). Using another syngeneic breast cancer cell line, 4T1, a similar metastasis-promoting effect was observed in 4T1-bearing mice treated with NAC (Figure S1A,B), although the primary tumors did not differ between the two groups (Figure S1C–E). Therefore, in immunocompetent mice, the administration of NAC promoted lung metastasis of breast cancer without affecting primary tumor growth.

### 2.2. NAC Does Not Affect the Malignant Behavior of Breast Tumor Cells In Vitro

**Figure 2:**
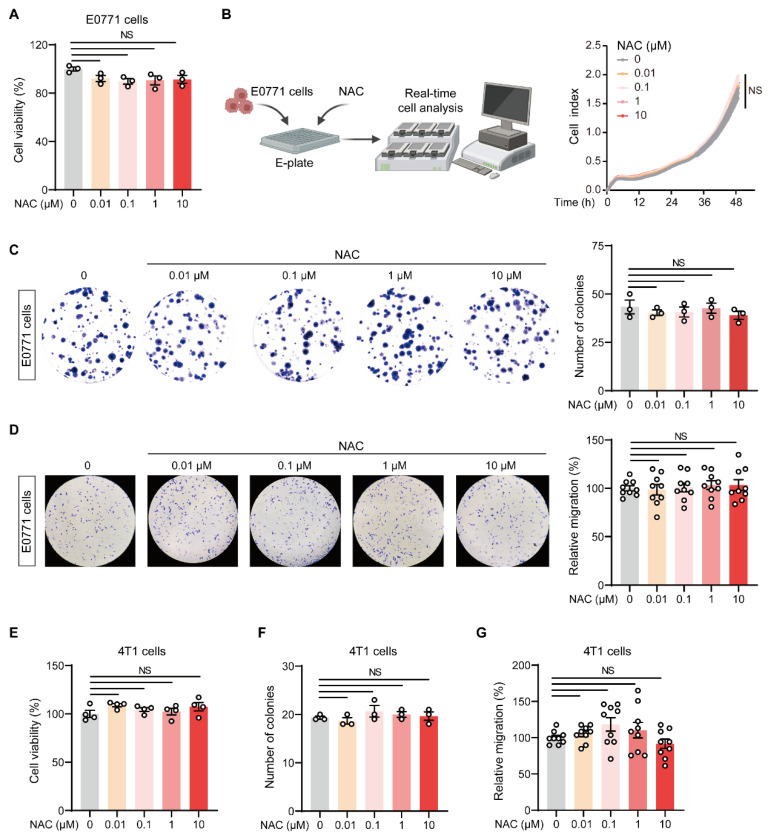
NAC does not affect the proliferation, clonogenic potential, or migration of breast cancer cells in vitro. (**A**) MTT assay was performed to measure the proliferation of E0771 cells (*n* = 3). The E0771 cells were seeded in 96-well plates (4 × 10^3^ cells/well) in the presence of vehicle or different concentrations of NAC (0.01–10 µM). Forty-eight hours later, the supernatant was removed, and the cells were supplemented with MTT solution (5 mg/mL). Following a 4-h incubation at 37 °C, the supernatant was aspirated and replaced with dimethyl sulfoxide (DMSO). The absorbance at 490 nm was measured. (**B**) Real-time cell analysis (RTCA) showed the proliferation rate of E0771 cells (*n* = 4). E0771 cells were seeded in an E-plate (4 × 10^3^ cells/well) in the presence of vehicle or different concentrations of NAC (0.01–10 µM). The E-plate was cultured for 48 h, and cell numbers were quantified with a real-time cell analyzer system. (**C**) The clonogenic potential of E0771 cells was assessed by seeding tumor cells in 6-well plates (200 cells/well) and culturing them in the presence of vehicle or different concentrations of NAC (0.01–10 µM). The plates were maintained for 12 days, and the colonies were fixed with methanol, stained with 0.5% crystal violet, and quantified (*n* = 3). (**D**) The migratory capacity of E0771 cells was measured. E0771 cells were first treated with vehicle or different concentrations of NAC (0.01–10 µM), then, 24 h later, the supernatant was removed, and the tumor cells were trypsinized to generate single-cell suspensions. Then, 2 × 10^5^ tumor cells were suspended in serum-free medium and loaded into the upper Transwell chamber. The lower chamber was supplemented with 500 µL of complete medium to serve as a chemoattractant. Twelve hours later, the cells in the upper chamber were fixed and stained with crystal violet. The tumor cells that migrated through the upper chamber were imaged and counted (*n* = 9). (**E**–**G**) The proliferation (**E**), clonogenic potential (**F**), or migration (**G**) of 4T1 cells were measured in the presence of vehicle or different concentrations of NAC (0.01–10 µM) using the same method as described in the legend for E0771 cells above (*n* = 4 in (**E**); *n* = 3 in (**F**); *n* = 9 in (**G**)). *n* represents the number of technical replicates. Data are shown as mean ± SEM, and statistical significance was determined using one-way ANOVA (**A**,**C**–**G**) or two-way ANOVA (**B**). NS, not significant.

We then determined whether NAC had a direct effect on in vitro-cultured breast tumor cells. Results from the 3-(4,5-dimethylthiazol-2-yl)-2,5-diphenyl tetrazolium bromide (MTT) assay (Figure 2A) and real-time cell analysis (RTCA) (Figure 2B) showed that the proliferation of NAC-treated E0771 cells was comparable with that of controls. Furthermore, NAC treatment did not alter the clonogenic potential (Figure 2C) or migration (Figure 2D) of E0771 cells. We also performed similar experiments using two other breast cancer cell lines, 4T1 and MDA-MB-231. Consistent with E0771 cells, NAC did not affect the proliferation (Figure 2E and Figure S2C), clonogenic potential (Figure 2F and Figure S2A,D), and migration (Figure 2G and Figure S2B,E) of these breast cancer cells in vitro. Collectively, NAC exhibited no direct impact on the malignant behavior of breast cancer cells, which raises the possibility that NAC promotes lung metastasis by modulating the immune microenvironment.

### 2.3. The Metastasis-Promoting Effect of NAC Is Abrogated in Immunodeficient Mice

**Figure 3:**
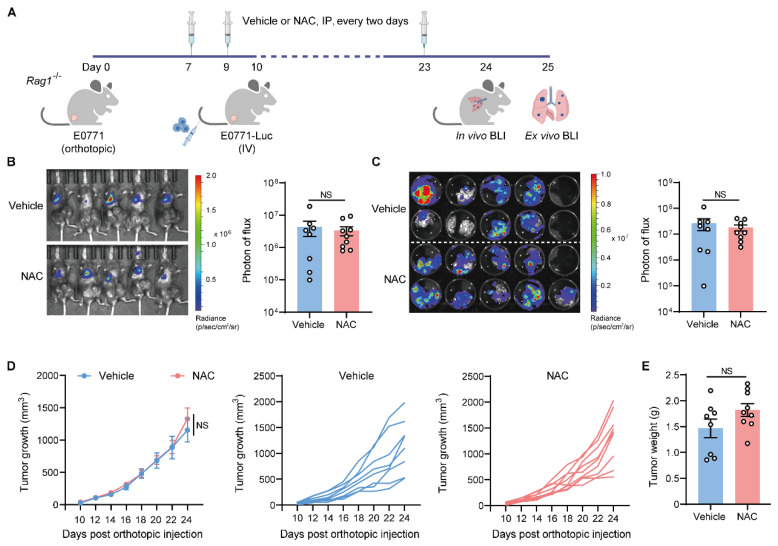
The metastasis-promoting effect of NAC is abrogated in T cell-deficient mice. (**A**–**D**) As depicted in the schematic (**A**), female *Rag1*^−/−^ mice first received an orthotopic inoculation of 2 × 10^5^ E0771 cells. From day 7, mice were IP-injected with vehicle or NAC (150 mg/kg) every 2 days. On day 10, E0771-Luc cells were IV-injected (1 × 10^6^ cells) into the tumor-bearing mice. On day 24, lung metastasis was quantified via in vivo BLI (**B**). On day 25, lung tissues were excised for ex vivo BLI (**C**). The primary tumor volume (**D**) was measured from day 10 to day 24 post the orthotopic implantation of E0771 cells, and tumor weight (**E**) was determined on day 25 (*n* = 8–9). *n* represents the number of biological replicates. Data are shown as mean ± SEM, and statistical significance was determined using the Mann–Whitney test (**B**,**C**,**E**) or two-way ANOVA (**D**). NS, not significant.

T cells are crucial players in the fight against cancer, capable of recognizing and destroying tumor cells. The dysfunction or exhaustion of T cells impairs immune surveillance, allowing tumor cells to avoid elimination and promote cancer growth and metastasis [24,25]. To define the role of T cells in NAC-modulated lung metastasis progression, we used *Rag1*^−/−^ mice, which are deficient in T and B cells, to conduct similar experiments (Figure 3A). Intriguingly, NAC treatment did not cause significant changes in lung metastatic colonization in *Rag1*^−/−^ mice (Figure 3B,C), and no effect was observed on primary tumor growth (Figure 3D,E). Thus, the metastasis-promoting effect of NAC was likely dependent on T cells.

### 2.4. NAC Does Not Affect the Proliferation and Effector Function of T Cells In Vitro

**Figure 4:**
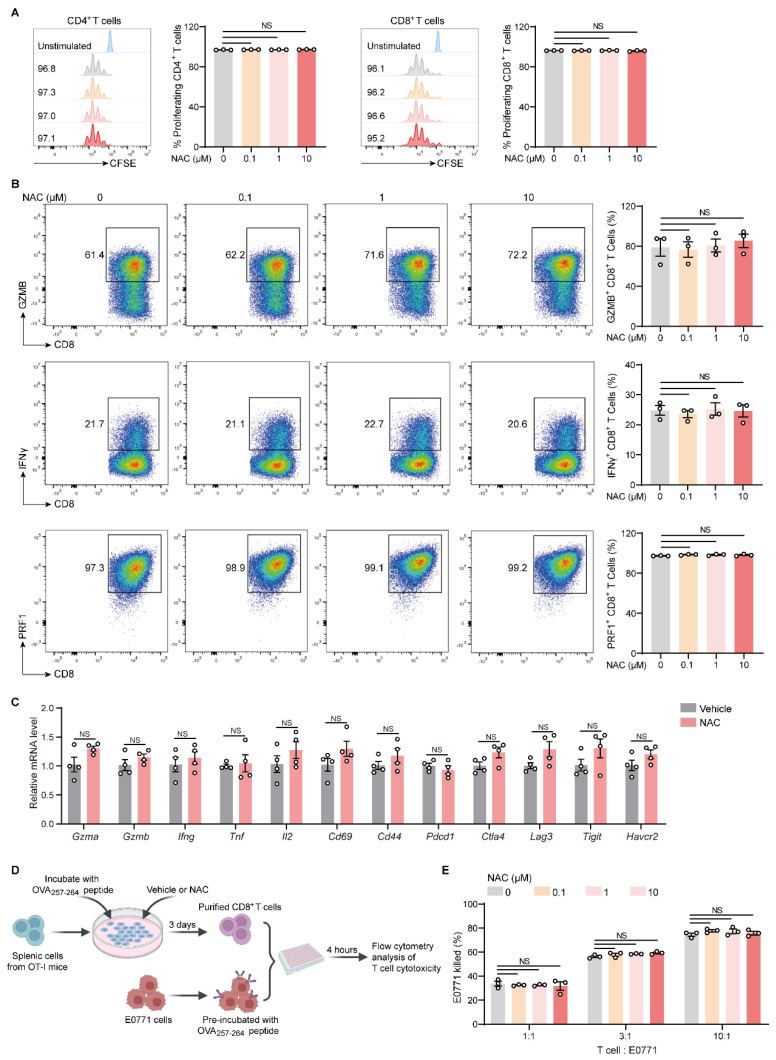
NAC does not affect the activation and function of T cells in vitro. (**A**) Splenic CD3^+^ T cells were purified from naive mice and labeled with 5-Carboxyfluorescein diacetate N-succinimidyl ester (CFSE) prior to ex vivo activation. Then, T cells were treated with vehicle or different concentrations of NAC (0.1–10 µM) in the presence of plate-coated anti-CD3 and soluble anti-CD28 for 72 h. T cell proliferation was measured via flow cytometry (*n* = 3). (**B**) Splenic CD8^+^ T cells were purified from naive mice and treated with vehicle or different concentrations of NAC (0.1–10 µM) in the presence of plate-coated anti-CD3 and soluble anti-CD28. Then, 72 h later, GolgiStop and GolgiPlug were added to the medium and incubated for 4 h. Then, T cells were subjected to intracellular cytokine staining to measure the frequencies of granzyme B (GZMB)^+^, interferon-gamma (IFNγ)^+^, and perforin-1 (PRF1)^+^ CD8^+^ T cells (*n* = 3). (**C**) Splenic CD8^+^ T cells were purified from naive mice and treated with vehicle or different concentrations of NAC (0.1–10 µM) in the presence of plate-coated anti-CD3 and soluble anti-CD28. Then, 72 h later, CD8^+^ T cells were harvested for RNA extraction. Next, total mRNA was transcribed to cDNA, and the expression of indicated genes in CD8^+^ T cells was determined by quantitative real-time PCR (*n* = 4). (**D**,**E**) As depicted in the schematic (**D**), splenic cells were harvested from OT-I mice and treated with vehicle or different concentrations of NAC (0.1–10 µM) in the presence of 1 μg/mL OVA_257–264_ peptide for 72 h. The CD8^+^ T cells were then isolated for further experiments. E0771 tumor cells were pre-treated with 1 μg/mL OVA_257–264_ peptide before washing. Then, the CD8^+^ T cells were co-cultured with E0771 cells (pre-loaded with OVA antigen) at different ratios (1:1, 3:1, 10:1) at 37 °C for 4 h. Dead tumor cells were measured with flow cytometry (**E**) (*n* = 3). *n* represents the number of biological replicates. Data are shown as mean ± SEM, and statistical significance was determined using one-way ANOVA (**A**,**B**,**E**) or unpaired Student’s *t* test (**C**). NS, not significant.

We further performed in vitro studies to investigate whether NAC directly alters T cells. The results showed that NAC treatment did not affect the proliferation of CD4^+^ and CD8^+^ T cells (Figure 4A). Additionally, the expression of anti-tumor cytokines, including granzyme B (GZMB), interferon-γ (IFNγ), and perforin (PRF1), in CD8^+^ T cells was not affected by NAC (Figure 4B). This was similarly observed (Figure S3A,B) when analyzing ex vivo activated OT-I CD8^+^ T cells. Further analysis of the mRNA levels of some effector molecules (*Gzma*, *Gzmb*, *Ifng*, *Tnf*), activation-associated genes (*Il2*, *Cd69*, *Cd44*), and exhaustion-associated genes (*Pdcd1*, *Ctla4*, *Lag3*, *Tigit*, *Havcr2*) in CD8^+^ T cells revealed that the expression of these genes did not differ between NAC-treated groups and controls (Figure 4C). Consistent with the above findings, the cytotoxicity of OT-I CD8^+^ T cells against antigen-pre-loaded E0771 cells was not affected by NAC (Figure 4D,E). Therefore, NAC does not directly impact the proliferation and effector function of T cells in vitro.

### 2.5. NAC Endows Neutrophils with Immunosuppressive Capacities Which Dampen the Activation and Function of T Cells

**Figure 5:**
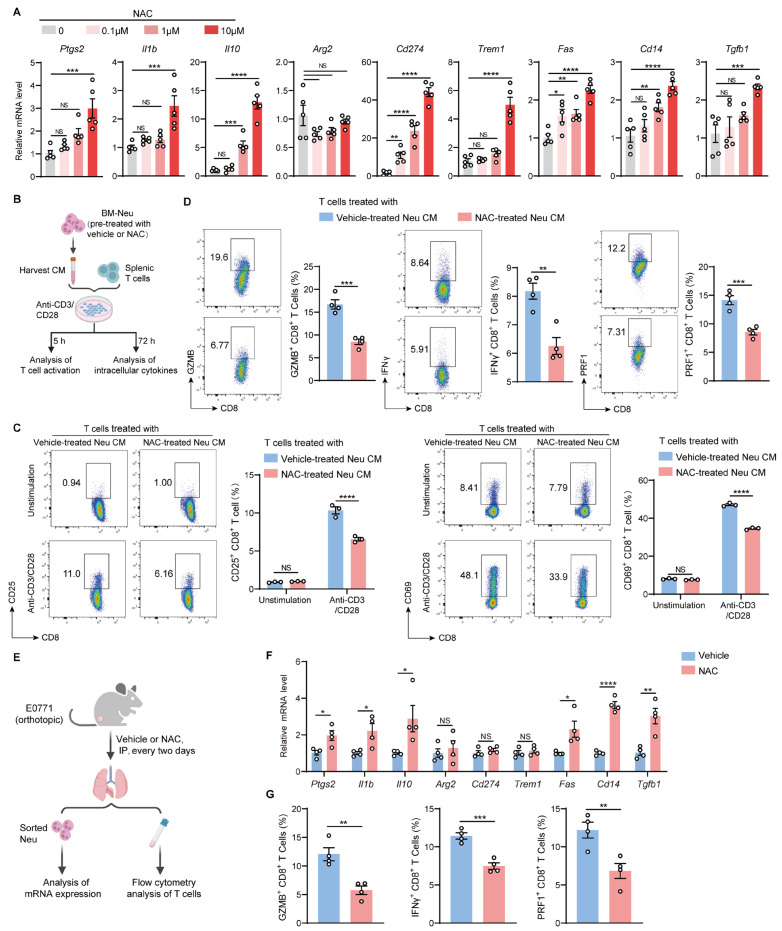
NAC reprograms neutrophils to be immunosuppressive. (**A**) BM-derived neutrophils were isolated from naïve mice using anti-Ly6G microbeads and treated with vehicle or different concentrations of NAC (0.1–10 µM) for 12 h and then harvested for RNA extraction. Total mRNA was transcribed to cDNA, and the expression of indicated genes was measured by RT-qPCR (*n* = 5). (**B**–**D**) As depicted in the schematic (**B**), BM-derived neutrophils were pre-treated with vehicle or 10 μM NAC and then cultured in RPMI-1640 medium for 12 h. The conditioned medium (CM) derived from vehicle-pre-treated or NAC-pre-treated neutrophils was collected and incubated with splenic T cells in the presence of plate-coated anti-CD3 and soluble anti-CD28, and 5 h later, T cells were harvested to measure the frequencies of CD25^+^CD8^+^ T cells and CD69^+^CD8^+^ T cells (**C**). Then, 72 h later, T cells were subjected to intracellular cytokine staining to measure the frequencies of GZMB^+^, IFNγ^+^, and PRF1^+^ CD8^+^ T cells (**D**) with flow cytometry (*n* = 3 in (**C**); *n* = 4 in (**D**)). (**E**–**G**) As depicted in the schematic (**E**), E0771 tumor-bearing mice were IP-injected with vehicle or NAC (150 mg/kg) every 2 days from day 7 to day 25. On day 26, lung tissues were harvested to generate single-cell suspensions. Then, neutrophils were sorted from crude lung cells and used to analyze the expression of indicated genes by RT-qPCR (**F**) (*n* = 4). Lung Ly6G^−^ cells were incubated with phorbol myristate acetate (PMA) (50 ng/mL, TargetMol), ionomycin (1 µg/mL, TargetMol), GolgiStop (1:1000, BD Biosciences), and GolgiPlug (1:1000, BD Biosciences) for 4 h at 37 °C. The frequencies of GZMB^+^, IFNγ^+^, and PRF1^+^ CD8^+^ T cells in the lung were measured with flow cytometry (**G**) (*n* = 4). *n* represents the number of biological replicates. Data are shown as mean ± SEM, and statistical significance was determined by one-way ANOVA (**A**) or unpaired Student’s *t* test (**C**,**D**,**F**,**G**). * *p* < 0.05, ** *p* < 0.01, *** *p* < 0.001, **** *p* < 0.0001; NS, not significant.

Myeloid-derived suppressor cells, including neutrophils and monocytes, are well-characterized components that facilitate solid tumor metastasis by impeding T cell function [26,27,28]. Given that NAC did not directly affect the activation and effector function of T cells, we hypothesized that NAC might modulate myeloid cells, which serve to suppress T cells. To address this, we isolated bone marrow (BM)-derived neutrophils or monocytes, cultured them with NAC, and analyzed the expression of immunosuppression-associated genes. The results showed that NAC significantly induced the expression of a series of immunosuppression-associated genes [29], including *Ptgs2*, *Il1b*, *Il10*, *Cd274*, *Trem1*, *Fas*, *Cd14*, and *Tgfb1*, in neutrophils (Figure 5A), while these genes were less affected in monocytes (Figure S4). Moreover, we collected the supernatants of NAC-treated neutrophils and performed functional assays. The expression of T cell activation markers (CD25 and CD69), as well as anti-tumor cytokines (GZMB, IFNγ, and PRF1), was significantly suppressed when incubated with the supernatants of NAC-pre-treated neutrophils (Figure 5B–D and Figure S5). In addition, certain immunosuppression-associated genes, including *Ptgs2*, *Il1b*, *Il10*, *Fas*, *Cd14*, and *Tgfb1*, were significantly upregulated in lung neutrophils from NAC-treated mice compared with controls (Figure 5E,F). Moreover, the frequencies of GZMB^+^, IFNγ^+^, and PRF1^+^ CD8^+^ T cells were reduced in the lungs of NAC-treated mice (Figure 5G). Taken together, NAC endows neutrophils with an immunosuppressive phenotype that represses T cell activation and function, likely through soluble factors.

## 3. Discussion

The role of NAC in cancer biology is multifaceted, but research has predominantly focused on its direct effects on tumor cells. In contrast to previous studies demonstrating that NAC can promote malignant behavior in certain tumor cells [15,17,30], our findings revealed that NAC had no significant impact on the proliferation, clonogenic potential, and migration of breast cancer cell lines, including E0771, 4T1, and MDA-MB-231. This inconsistency highlights the complex and context-dependent effects of antioxidants on cancer cells. The primary factor contributing to this divergence is likely the intrinsic genetic and molecular background of the tumor cell line. The pro-tumorigenic effects of NAC observed across different cancer cells might depend on distinct oncogenic properties and cellular redox states. For instance, tumor cells with intact p53 function or low basal reliance on ROS for survival are more sensitive to antioxidant perturbation [31,32]. Given that breast cancer cells are highly heterogeneous with distinct genetic and non-genetic backgrounds [33], the cell lines that we used in this study might be intrinsically resistant to NAC stimulation. Additionally, the concentration-dependent effects of antioxidants represent a critical consideration. Previous research has demonstrated that low concentrations (5–25 µM) of vitamin C enhance the proliferation of colon cancer stem cells (CSCs), whereas high concentrations (100–1000 µM) exhibit cytotoxicity against colon CSCs [34]. Therefore, it is possible that the NAC concentrations (0.01–10 µM) used in the in vitro study were insufficient to elicit the downstream signaling cascades that are required to drive malignant phenotypes. Future studies involving a more comprehensive dose–response analysis will be essential to investigate the exact effects of NAC on breast cancer cells.

In our study, we employed an experimental lung metastasis model wherein tumor cells were directly injected into the venous circulation. While this approach does not model the initial steps of metastasis, such as detachment from the primary tumor and intravasation, it is a well-validated method for specifically investigating the later colonization phase, where circulating tumor cells extravasate, survive, and proliferate within the distant organ microenvironment. This model was chosen as it allows for controlled and quantitative assessment of the lung metastatic burden, which is central in evaluating the effects of NAC at this specific stage. Future studies utilizing spontaneous metastasis models from orthotopic primary tumors would be helpful in confirming the impact of NAC on the complete metastatic cascade.

Neutrophils, once considered short-lived effector cells, are now recognized as critical regulators of tumor progression and metastasis [35,36]. The pro-tumorigenic functions of neutrophils are mediated through multiple mechanisms. For example, they potently restrain anti-tumor immunity by releasing immunosuppressive factors such as IL-10 and TGF-β1, generating ROS, and expressing immune checkpoint ligands to inhibit the effector function of T cells and NK cells [37,38,39,40]. In addition, in the metastatic cascade, neutrophils facilitate invasion and distant seeding by forming neutrophil extracellular traps (NETs), which capture circulating tumor cells, degrade the extracellular matrix, and awaken dormant metastatic cells [41,42,43,44]. In our study, we showed that NAC endow neutrophils with an immunosuppressive phenotype, which is characterized by the upregulation of immunosuppressive genes, and that NAC-educated neutrophils potently suppress the activation and effector function of T cells. Although other studies have reported different effects of NAC on neutrophils, including the inhibition of chemotaxis, ROS production, and NET formation [45,46], the direct induction of immunosuppressive capacities by NAC has not been demonstrated. Our results suggest that NAC does not merely attenuate neutrophil function but promotes a functional shift toward an immunosuppressive phenotype, thereby providing novel insights into the immunomodulatory effects of NAC. However, the underlying mechanism behind this phenomenon requires further investigation. It is plausible that by altering intracellular redox balance and GSH levels, NAC triggers transcriptional reprogramming in neutrophils, which ultimately leads to the upregulation of immunosuppressive genes. Future efforts could focus on delineating the specific molecular and signaling pathways responsible for this functional switch.

## 4. Materials and Methods

### 4.1. Animals

C57BL/6, BALB/c, and *Rag1*^−/−^ mice were acquired from GemPharmatech (Jiangsu, China). All mice were housed under a 12-h light/dark cycle at 22–24 °C with 50–60% humidity and maintained in the same pathogen-free conditions. Mice aged 8–10 weeks were randomly selected for each experiment. The sample size for animal experiments was determined based on the level of expected heterogeneity of the samples, the significance threshold (chosen at 0.05), and the expected or observed difference, as well as our pilot studies for each experiment. All experiments were unblinded because these analyses were performed using quantifiable parameters, and no bias was involved. All animal experiments were performed in accordance with the guidelines from the Experimental Animal Welfare and Ethics Committee of the Institute of Health and Medicine, Hefei Comprehensive National Science Center. The related experimental protocols are approved with the approval number IHM-AP-2024-058.

### 4.2. Cell Culture

The following breast cancer cell lines were selected for in vitro studies: E0771 (a C57BL/6 mouse-derived mammary adenocarcinoma cell line), 4T1 (a highly metastatic triple-negative cell line from BALB/c mice), and MDA-MB-231 (a human triple-negative breast cancer cell line). This panel was chosen for its complementary roles in evaluating the effects of NAC on the malignant behavior of breast cancer cells across different species and genetic backgrounds. The 4T1 cell line was obtained from FuHeng Biology (Shanghai, China). The MDA-MB-231 cell line was obtained from Immocell Biotechnology (Amoy, China). The E0771 cell line was kindly provided by Dr. Hanqiu Zheng (Tsinghua University). The 4T1 cells were grown in RPMI-1640 medium plus 10% fetal bovine serum (FBS) and 1% penicillin/streptomycin (P/S). MDA-MB-231 cells and E0771 cells were cultured in DMEM medium plus 10% FBS and 1% P/S. Cell lines were validated through short tandem repeat analysis and routinely cultured in an incubator at 37 °C with 5% CO_2_. To generate luciferase-labeled tumor cells, the luciferase gene was transduced into E0771 and 4T1 cells via a lentiviral system to establish stable expression.

### 4.3. Generation of Luciferase-Labeled Tumor Cells

For the production of lentiviral, HEK293T cells were seeded in 10 cm dishes (2 × 10^6^ cells per dish) one day prior to transfection. 2.5 µg of pMD2.G envelope plasmid (Addgene plasmid #12259), 7.5 µg of psPAX2 packaging plasmid (Addgene plasmid #12260), and 10 µg of the luciferase-expressing lentiviral plasmid (Addgene plasmid #17477) were co-transfected into the HEK293T cells using Lipofectamine 3000 reagent (Invitrogen, Carlsbad, CA, USA) according to the manufacturer’s protocol. At 48 h post-transfection, virus particles were collected, centrifuged at 300× *g* for 5 min to remove cellular debris, filtered through a 0.45 µm filter, and stored at −80 °C.

For viral transduction, tumor cells were seeded in 6-well plates (2 × 10^5^ cells per well) 24 h before infection. Then tumor cells were incubated with virus supernatant, which was diluted 1:1 in DMEM medium supplemented with 5 µg/mL polybrene (Sigma-Aldrich, St. Louis, MO, USA) to enhance transduction efficiency. After 24 h, the medium was replaced with fresh culture medium containing 4 µg/mL puromycin (Solarbio, Shanghai, China) for selection. Puromycin-resistant cells were maintained under selection pressure for 5–7 days until no cell death was observed, thus establishing stable luciferase-expressing cell lines.

### 4.4. Breast Cancer Metastasis Models

To evaluate the effect of NAC on lung metastasis progression, female mice first received an orthotopic inoculation of 2 × 10^5^ breast cancer cells (E0771 or 4T1). On day 7, mice were intraperitoneally (IP) injected with vehicle or NAC (150 mg/kg) every 2 days. On day 10, luciferase-labeled E0771 or 4T1 cells were intravenously (IV) injected (1 × 10^6^ cells) into the tumor-bearing mice. Lung metastasis was quantified by measuring bioluminescent signal intensity. For the primary tumor, measurements were taken using a caliper, and volume (mm^3^) was calculated as (length  ×  width^2^)/2.

Unless otherwise stated, anesthetized mice received an IP injection of D-luciferin (150 mg/kg) for in vivo BLI. For ex vivo BLI of lung tissues, the mice were sacrificed at the endpoint, and the lungs were excised and placed in a plate supplemented with D-luciferin (300 µg/mL). BLI data were acquired with the IVIS imaging system (PerkinElmer, Waltham, MA, USA), and signal intensity in the region of interest was quantified using Living Image software version 4.2 (PerkinElmer, Waltham, MA, USA).

### 4.5. Cell Proliferation Assay

The MTT assay was carried out to evaluate the effect of NAC on the proliferation of different tumor cells (E0771, 4T1, and MDA-MB-231). The tumor cells were trypsinized and seeded in 96-well plates (4 × 10^3^ cells/well) in the presence of different concentrations of NAC. Forty-eight hours later, the supernatant was removed, and the cells were supplemented with MTT solution (5 mg/mL). Following a 4-h incubation at 37 °C, the supernatant was carefully aspirated and replaced with DMSO. The plates were shaken for 15 min, and the absorbance at 490 nm was measured with a microplate reader (PE Ensight, PerkinElmer).

For the real-time cell analysis (RTCA) of the tumor cell proliferation, E0771 cells were seeded in an E-plate (300600910, Agilent) (4 × 10^3^ cells/well) in the presence of vehicle or NAC. The E-plate was cultured for 48 h, and cell numbers were quantified with the real-time cell analyzer system (Agilent, Santa Clara, CA, USA).

### 4.6. Colony Formation Assay

Clonogenic potential was assessed by seeding tumor cells in 6-well plates (200 cells/well) and culturing them in the presence of vehicle or NAC. The plates were maintained for 12 days, and the colonies were fixed with methanol, stained with 0.5% crystal violet, and quantified.

### 4.7. Transwell Migration Assay

Tumor cells were first treated with vehicle or different concentrations of NAC. 24 h later, the supernatant was removed, and the tumor cells were trypsinized to generate single-cell suspensions. Next, 2 × 10^5^ tumor cells were suspended in serum-free medium and loaded into the upper Transwell chamber. The lower chamber was supplemented with 500 µL of complete medium to serve as a chemoattractant. Twelve hours later, the cells in the upper chamber were fixed and stained with crystal violet. The tumor cells that migrated through the upper chamber were imaged and counted.

### 4.8. Lung Tissue Dissociation

Tissue dissociation was conducted as described previously [47]. The mice were sacrificed and perfused with PBS. Lungs were infused with enzyme solution containing collagenase type IV (2 mg/mL, Worthington Biochemical, Lakewood, NJ, USA) and DNase I (20 μg/mL, Sigma-Aldrich, St. Louis, MO, USA). The lung tissues were then harvested, minced into small pieces, and enzymatically dissociated at 37 °C for 50 min. The cell suspensions were passed through 100-μm strainers and subjected to erythrocyte lysis with ammonium chloride potassium (ACK) buffer.

### 4.9. Primary Cell Isolation

Bone marrow cells were flushed from the tibias and femurs of mice. The harvested cells were subsequently subjected to erythrocyte lysis with ACK buffer and filtered through 40-μm strainers. Then, the neutrophils were purified from bone marrow cells using anti-Ly6G microbeads (Miltenyi Biotech, Bergisch Gladbach, NRW, Germany). Spleens were carefully excised and mechanically dissociated using syringe plungers. The cell suspension was then passed through 70-μm strainers and subjected to erythrocyte lysis with ACK buffer. CD3^+^ T cells were purified from splenic cells using CD90.2 microbeads (Miltenyi Biotec, Bergisch Gladbach, NRW, Germany) following the manufacturer’s instructions.

### 4.10. Flow Cytometry

Single-cell suspensions were resuspended in fluorescence-activated cell sorting (FACS) buffer (PBS plus 2% FBS) and subjected to surface marker staining with fluorochrome-conjugated antibodies for 30 min at 4 °C. Prior to analysis, 4,6-diamidino-2-phenylindole (DAPI) was added to stain dead cells. For intracellular cytokine staining (ICS) of lung T cells, the procedure was performed as described previously [48]. Briefly, neutrophils were depleted from lung crude cells with anti-Ly6G MicroBeads to avoid potential artifacts, and lung Ly6G^−^ cells were incubated with PMA (50 ng/mL, TargetMol, Boston, MA, USA), ionomycin (1 µg/mL, TargetMol, Boston, MA, USA), GolgiStop (1:1000, BD Biosciences, San Jose, CA, USA), and GolgiPlug (1:1000, BD Biosciences, San Jose, CA, USA) for 4 h at 37 °C. For the ICS of ex vivo activated T cells, GolgiStop and GolgiPlug were added to the medium 4 h before harvest. The Live/Dead Fixable Blue Dye (L34962, Thermo Fisher Scientific, Waltham, MA, USA) was used to label dead cells, and cells were then stained with surface primary antibodies for 30 min at 4 °C. Cells were then fixed and permeabilized using the Fixation/Permeabilization Solution Kit (554715, BD Biosciences, San Jose, CA, USA) and stained with fluorochrome-conjugated antibodies of intracellular cytokines (GZMB, IFNγ, and PRF1). Flow cytometry was performed with Cytoflex LX (Beckman, Brea, CA, USA).

### 4.11. T Cell Function Analysis

CD3^+^ T cells were purified from the spleens of naive mice and stimulated with plate-coated anti-CD3 (Bio X cell, Lebanon, NH, USA) and soluble anti-CD28 (Bio X cell, Lebanon, NH, USA). For the experiments with OT-I CD8^+^ T cells, splenic cells were harvested from OT-I mice and incubated with OVA_257–264_ peptide (vac-sin, InvivoGen, San Diego, CA, USA). To measure the effect of NAC on T cells, different concentrations of NAC (0, 0.1 μM, 1 μM, 10 μM) were added to the medium, and 3 days later, the T cells were harvested for functional analysis. To measure the impact of Neu-derived CM on T cells, BM-derived neutrophils were pre-treated with vehicle or 10 μM NAC and cultured in RPMI-1640 medium for 12 h. The conditioned medium was collected and incubated with T cells. To measure T cell proliferation, 5-Carboxyfluorescein diacetate N-succinimidyl ester (CFSE, MedChemExpress, Monmouth Junction, NJ, USA) was used to label T cells prior to ex vivo activation. To measure T cell activation, T cells were cultured for 5 h and then stained with anti-CD25 and anti-CD69. To assess the expression of intracellular cytokines, T cells were activated for 72 h and then subjected to ICS.

### 4.12. In Vitro T Cell Cytotoxicity Assay

Splenic cells were harvested from OT-I mice and treated with vehicle or different concentrations of NAC in the presence of 1 μg/mL OVA_257–264_ peptide (InvivoGen, San Diego, CA, USA) for 72 h. The CD8^+^ T cells were then isolated for further experiments. E0771 tumor cells were pre-treated with 1 μg/mL OVA_257–264_ peptide before washing. Then, the CD8^+^ T cells were co-cultured with the E0771 cells (pre-loaded with OVA antigen) at different ratios (1:1, 3:1, 10:1) at 37 °C for 4 h. Dead tumor cells were measured with flow cytometry.

### 4.13. RNA Extraction and Quantitative Real-Time PCR

Total mRNA was extracted from T cells, neutrophils, or monocytes using the RNA extraction kit (TIANGEN Biotech, Beijing, China) according to the manufacturer’s protocol. cDNA was generated using the cDNA Reverse Transcription kit (4368814, Thermo Fisher Scientific, Waltham, MA, USA). Quantitative real-time PCR (RT-qPCR) was then carried out using SYBR Green Mix (11184ES08, Yeasen Biotechnology, Shanghai, China) on the Light Cycler 480 (Roche, Basel, Basel-Stadt, Switzerland). Gene expression was analyzed with 2^−ΔΔCt^, and the housekeeping gene was *Rps18*. The primers used in this study are listed in Table S1.

### 4.14. Illustration Tool

The schematic images were created in BioRender. Li, Q. (2026) https://BioRender.com/i1rj8l2 (accessed on 26 December 2025).

### 4.15. Statistical Analyses

The results are presented as the mean ± standard error of the mean (SEM). For comparisons between two groups, statistical significance was determined using an unpaired Student’s *t*-test or a Mann–Whitney test. For comparisons across multiple groups, statistical significance was determined using one-way ANOVA or two-way ANOVA. All analyses were performed using GraphPad Prism version 8.4.2 (GraphPad Software Inc., San Diego, CA, USA). Statistical significances are indicated using asterisks: * *p* < 0.05, ** *p* < 0.01, *** *p* < 0.001, **** *p* < 0.0001. Non-significant outcomes are denoted as NS.

## 5. Conclusions

Our findings elucidate a previously unrecognized role played by NAC in modulating innate immune cells. These insights into the ability of NAC to drive the immunosuppressive reprogramming of neutrophils offer a novel perspective on its biological activities. This study enhances our understanding of how antioxidant supplements may affect cancer progression by reshaping the immune microenvironment, identifies a novel immunoregulatory function of NAC, and highlights the necessity of considering immune-mediated mechanisms when evaluating the systemic impact of antioxidant agents in cancer patients.

## Data Availability

The original contributions presented in this study are included in the article/Supplementary Materials. Further inquiries can be directed to the corresponding authors.

## Supplementary Materials

The following supporting information can be downloaded at: https://www.mdpi.com/article/10.3390/ijms27010526/s1.
