# Supplementary material for: Antioxidant N-Acetylcysteine Facilitates Breast Cancer Metas-Tasis via Immunosuppressive Reprogramming of Neutrophils
Source: Int J Mol Sci. 2026 Jan 4;27(1):526. doi: 10.3390/ijms27010526 (PMC12787033; doi:10.3390/ijms27010526)
Supplement: Supplementary file 1 [file ijms-27-00526-s001.zip › ijms-4031073-supplementary.pdf]

Table S1. Sequences of the primers used for RT-qPCR

Gene	Sequence
Rps18	F: GGAGAACTCACGGAGGATGA<br>R: CCAGTGGTCTTGGTGTGCTG
Gzma	F: GGGGCTCACTCAATCAATAAGG<br>R: CATCCTGCTACTCGGCATCT
Gzmb	F: GAAGCCAGGAGATGTGTGCT<br>R: GCACGTTTGGTCTTTGGGTC
Ifng	F:<br>GGCCATCAGCAACAACATAAGCGT<br>R: TGGGTTGTTGACCTCAAACTTGGC
Tnf	F: ACCCTCACACTCACAAACCA<br>R: ATAGCAAATCGGCTGACGGT
Il2	F: TGAAACTCCCCAGGATGCTC<br>R: AAAGTCCACCACAGTTGCTGA
Cd69	F: CCCTTGGGCTGTGTTAATAGTG<br>R: AACTTCTCGTACAAGCCTGGG
Cd44	F: TCGATTTGAATGTAACCTGCCG<br>R: CAGTCCGGGAGATACTGTAGC
Pdcd1	F: CGGTTTCAAGGCATGGTCATTGG<br>R: TCAGAGTGTCGTCCTTGCTTCC
Ctla4	F: TTTTGTAGCCCTGCTCACTCT<br>R: CTGAAGGTTGGGTCACCTGTA
Lag3	F: CCTCGATGATTGCTAGTCCCT<br>R: GTAGACAGGCACTCGGTTCTG
Tigit	F: GAATGGAACCTGAGGAGTCTCT<br>R: AGCAATGAAGCTCTCTAGGCT
Havcr2	F: TCAGGTCTTACCCTCAACTGTG<br>R: GGCATTCTTACCAACCTCAAACA
Ptgs2	F: CAGACAACATAAACTGCGCCTT<br>R: GATACACCTCTCCACCAATGACC
Il1b	F: ACCTTCCAGGATGAGGACATGA<br>R: CTAATGGGAACGTCACACACCA
Il10	F: CCAAGCCTTATCGGAAATGA<br>R: TTTTCACAGGGGAGAAATCG
Arg2	F: CACCTCTCACCACTGTATCTGG<br>R: CCAGGAAAATCCTGGCAGTTGTG
Cd274	F: GCTCCAAAGGACTTGTACGTG<br>R: TGATCTGAAGGGCAGCATTTC
Trem1	F: AGTCGTTGGAGCTGAGCTTG<br>R: CCAGGGTCAAGGGTTCCTTC
Fas	F: CTGCGATTCTCCTGGCTGTGAA

	R: CAACAACCATAGGCGATTTCTGG
Cd14	F: CTCTGTCCTTAAAGCGGCTTAC<br>R: GTTGCGGAGGTTCAAGATGTT
Tgfb1	F: TCACTGGAGTTGTACGGCAGTG<br>R: TCGAAAGCCCTGTATTCCGTC

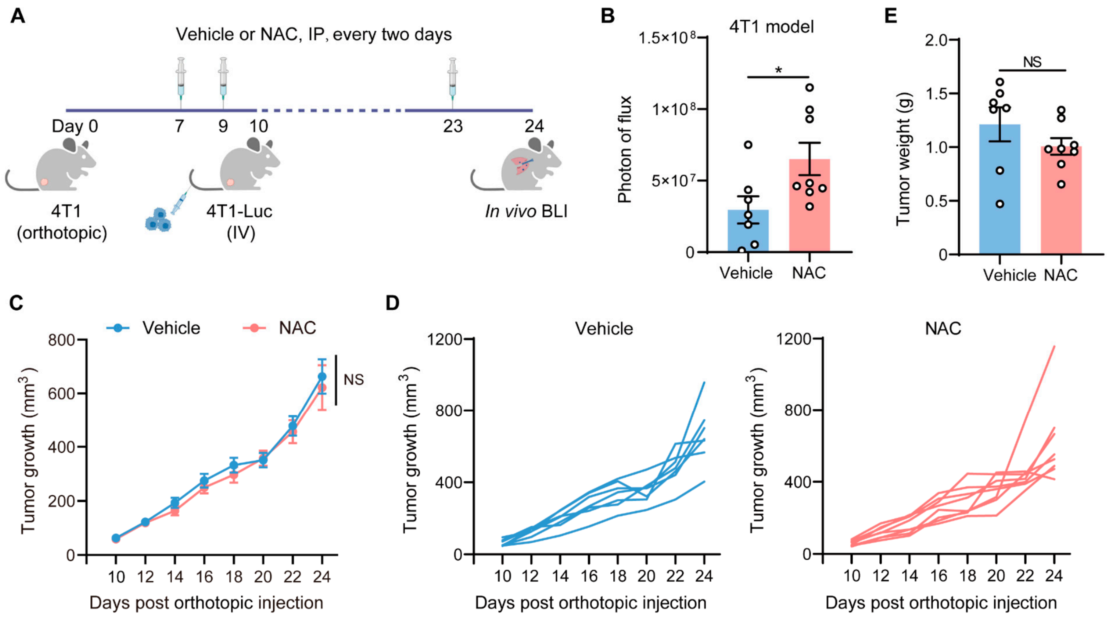

Figure S1. The effects of NAC on the experimental lung metastasis model of 4T1-bearing mice.

(A-E) As depicted in the schematic (A), female mice first received an orthotopic inoculation of 2×10^5 4T1 cells. From day 7, mice were intra-peritoneally (IP) injected with vehicle or NAC (150 mg/kg) every 2 days. On day 10, the luciferase-labeled 4T1 cells (4T1-Luc) were intravenously (IV) injected (1×10^6 cells) into the tumor-bearing mice. On day 24, lung metastasis was quantified by *in vivo* BLI (B). The primary tumor volume (C, D) was measured from day 10 to day 24 post the orthotopic implantation of 4T1 cells, and tumor weight (E) was determined on day 25 (*n*=7 - 8).

n represents the number of biological replicates. Data are shown as mean±SEM, and statistical significance was determined by the Mann–Whitney test (B and E) or two-way ANOVA (C). *$p < 0.05$; NS, not significant.

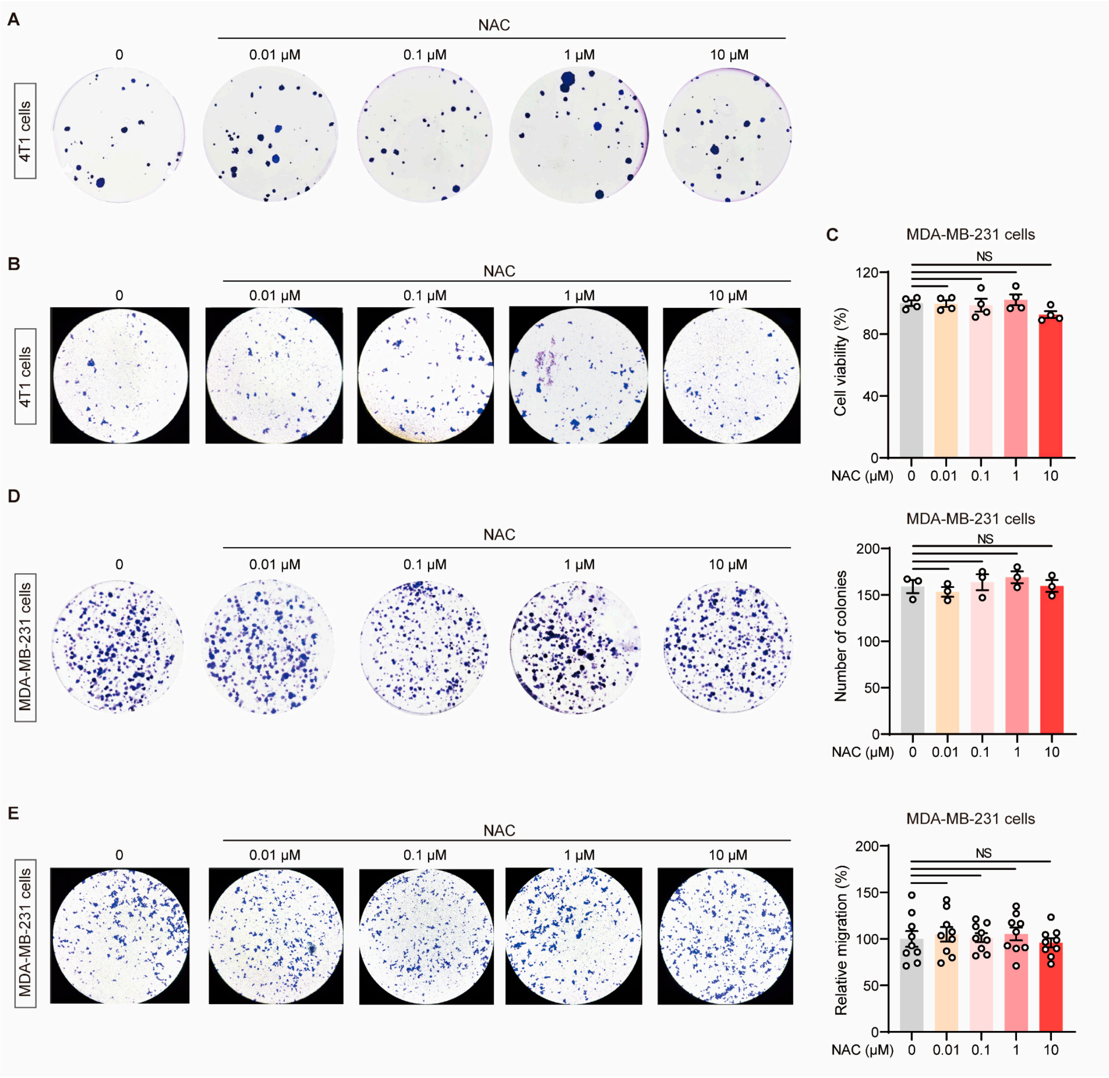

Figure S2. The effects of NAC on *in vitro* cultured 4T1 cells and MDA-MB-231 cells.

(A-B) Representative photographs of the colony-formation assay (A) or migration assay (B) of 4T1 cells in the presence of vehicle or different concentrations of NAC (0.01 – 10 μM). The experimental methods were as described in the legends for Figure 2F and 2G.

(C-E) Using the same methods as described for E0771 cells (Figure 2), the proliferation (C), clonogenic potential (D), or migration (E) of MDA-MB-231 cells was measured in the presence of vehicle or different concentrations of NAC (0.01 – 10 μM) (n=4 in C; n=3 in D; n=9 in E). Representative photographs of the colony-formation assay (D) or migration assay (E) of MDA-MB-231 cells were shown.

n represents the number of technical replicates. Data are shown as mean±SEM, and statistical significance was determined by one-way ANOVA (C-E). NS, not significant.

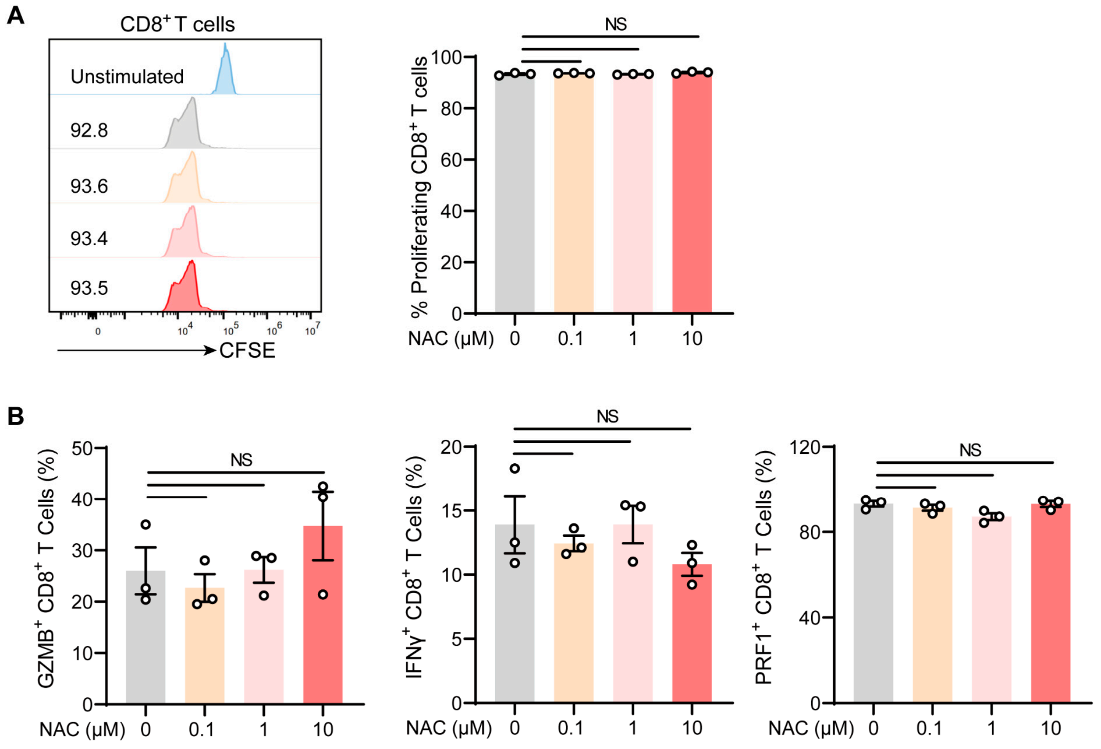

Figure S3. NAC does not affect the proliferation and function of OT-I CD8⁺ T cells *in vitro*.

(A-B) Splenic cells were isolated from OT-I mice and labeled with 5-Carboxyfluorescein diacetate N-succinimidyl ester (CFSE) prior to *ex vivo* activation. Then splenic cells were treated with vehicle or different concentrations of NAC (0.1 – 10 μM) in the presence of $OVA_{257-264}$ peptide (1 μg/ml). 72 hours later, GolgiStop and GolgiPlug were added to the medium and incubated for 4 h. The proliferation of $CD8^+$ T cells (A) and the frequencies of $GZMB^+$, $IFN\gamma^+$, and $PRF1^+$ $CD8^+$ T cells were measured by flow cytometry (B) (*n*=3).

n represents the number of biological replicates. Data are shown as mean±SEM, and statistical significance was determined by one-way ANOVA (A and B). NS, not significant.

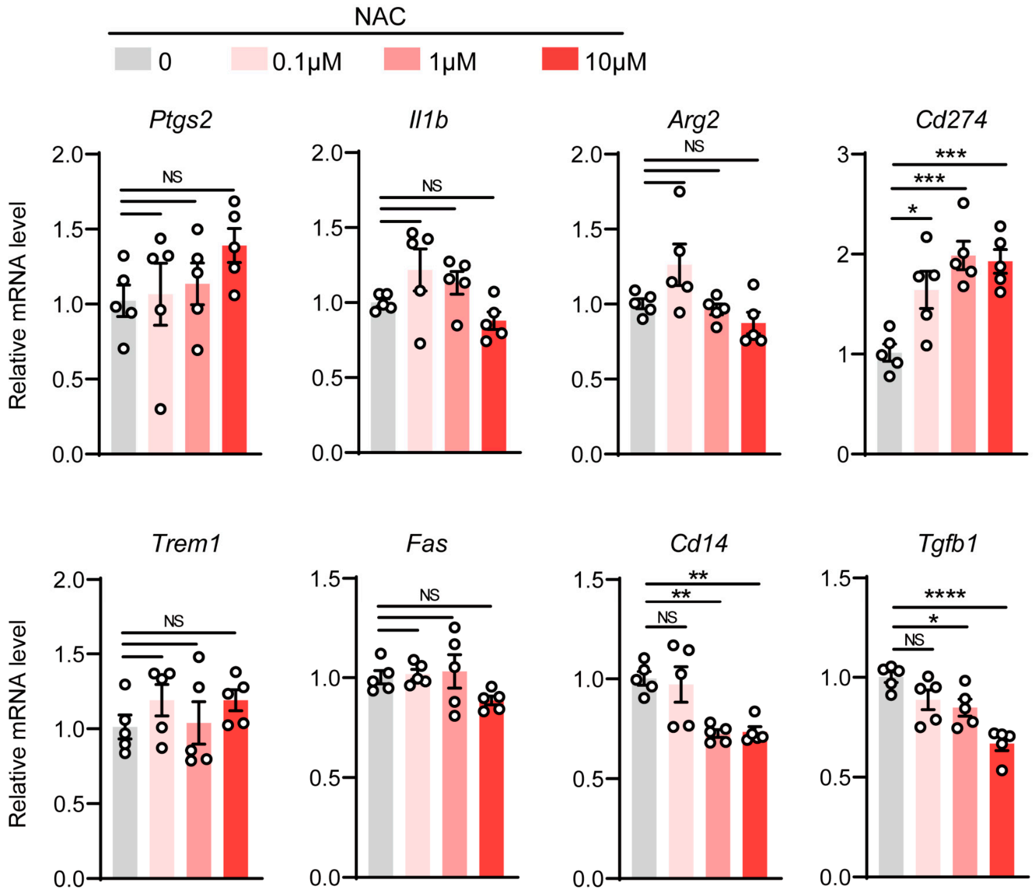

Figure S4. The effects of NAC on BM-derived monocytes.

BM-derived monocytes were sorted from BM cells of naïve mice and treated with vehicle or different concentrations of NAC (0.1 – 10 μM) for 12 hours, then the monocytes were harvested for RNA extraction. Total mRNA was transcribed to cDNA, and the expression of indicated genes was measured by RT-qPCR (*n*=5).

n represents the number of biological replicates. Data are shown as mean±SEM, and statistical significance was determined by one-way ANOVA. $^{*}p < 0.05$, $^{**}p < 0.01$, $^{***}p < 0.001$, $^{****}p < 0.0001$; NS, not significant.

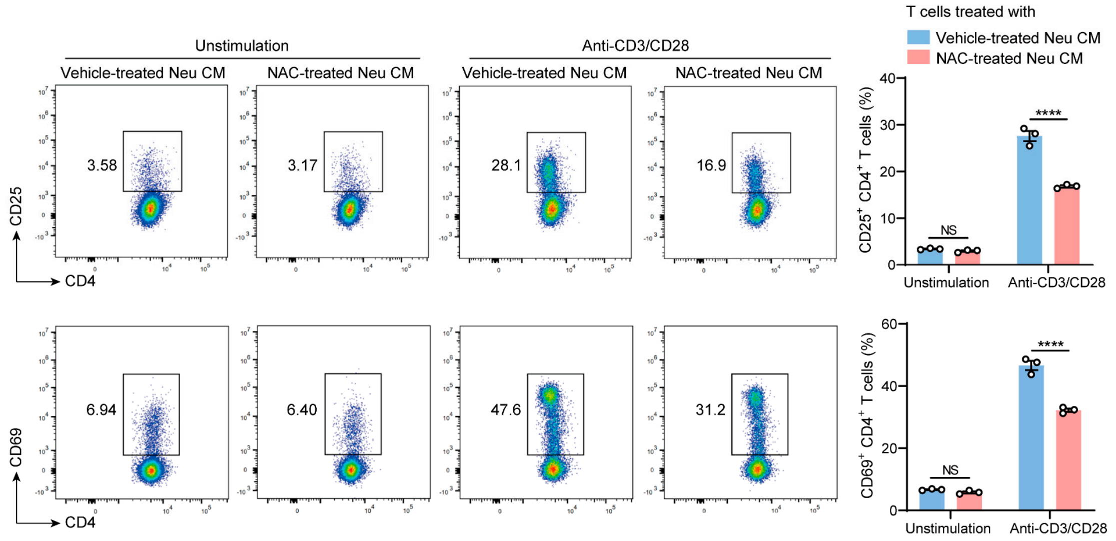

Figure S5. NAC-educated neutrophils suppress the activation of T cells.

As depicted in the schematic in Figure 5B, BM-derived neutrophils were pre-treated with vehicle or 10 μM NAC, then the neutrophils were cultured in RPMI-1640 medium for 12 hours. The conditioned medium (CM) derived from vehicle-pre-treated or NAC-pre-treated neutrophils was collected and incubated with splenic T cells in the presence of plate-coated anti-CD3 and soluble anti-CD28, and 5 hours later, T cells were harvested to measure the frequencies of $CD25^+CD4^+$ T cells (top) and $CD69^+CD4^+$ T cells (bottom) with flow cytometry (*n*=3).

n represents the number of biological replicates. Data are shown as mean±SEM, and statistical significance was determined by an unpaired Student's t-test. ****p < 0.0001; NS, not significant.
